# Potential Application of Lactic Acid Bacteria to Reduce Aflatoxin B_1_ and Fumonisin B_1_ Occurrence on Corn Kernels and Corn Ears

**DOI:** 10.3390/toxins12010021

**Published:** 2019-12-31

**Authors:** Tiago de Melo Nazareth, Carlos Luz, Raquel Torrijos, Juan Manuel Quiles, Fernando Bittencourt Luciano, Jordi Mañes, Giuseppe Meca

**Affiliations:** 1 Laboratory of Food Chemistry and Toxicology, Faculty of Pharmacy, University of Valencia, Av. Vicent Andrés Estellés s/n, 46100 Burjassot, Spain; carlos.luz@uv.es (C.L.); raquel.torrijos@uv.es (R.T.); juan.quiles@uv.es (J.M.Q.); jorge.manes@uv.es (J.M.); 2 School of Life Sciences, Pontifícia Universidade Católica do Paraná, st. Imaculada Conceição 1155, Curitiba 80215-901, PR, Brazil; fernando.luciano@pucpr.br

**Keywords:** *Lactobacillus plantarum*, aflatoxin B_1_, fumonisin B_1_, biopreservation

## Abstract

Fungal spoilage is an important issue for the food industry, leading to food sensory defects, food waste, economic losses and public health concern through the production of mycotoxins. Concomitantly, the search for safer natural products has gained importance since consumers began to look for less processed and chemically treated foods. In this context, the aim of this study was to evaluate the antifungal and antimycotoxigenic effect of seven strains of *Lactobacillus plantarum*. Lactic acid bacteria (LAB) were grown on Man Rogosa Sharpe (MRS) broth at 37 °C in anaerobic conditions. After that, the cell-free supernatant (CFS) were recovered to determine its antifungal activity by halo diffusion agar test. In addition, minimum inhibitory concentration (MIC) and minimum fungicidal concentration (MFC) was determined for each *L. plantarum* CFS by 96-well microplates method. Additionally, CFS was used as a natural biocontrol agent on corn kernels and corn ears contaminated with *Aspergillus flavus* and *Fusarium verticillioides*, respectively. The *L. plantarum* CECT 749 CFS showed the highest antifungal effect against all essayed strains. Moreover, the employment of this CFS in food reduced the mycotoxin production at a percentage ranging from 73.7 to 99.7%. These results suggest that the *L. plantarum* CECT 749 CFS could be promising for the biocontrol of corn.

## 1. Introduction

Filamentous fungi contaminate several types of food such as cereals, fruits and vegetables, dried fruits, feed products, dried spices, dried cured meats, and bread, among others [1,2,3,4,5,6,7,8,9].

*Aspergillus* and *Fusarium* are the major fungal genera associated with corn contamination [10]. Indeed, many *Aspergillus* and *Fusarium* species are considered common contaminants of food and feed due to their ability to colonize and grow in a wide range of environmental conditions. Furthermore, these species are also known to produce mycotoxins.

Mycotoxins are secondary metabolites produced by the mycelial structure of filamentous fungi that have no biochemical significance in fungal growth but have the potential to elicit undesirable effects on human and animal health, following consumption of contaminated food or feedstuffs [11,12,13]. Therefore, mycotoxin contaminated crops result in illness and economic losses once these grains cannot be ingested or marketed.

Several factors can influence the presence of mycotoxin in foods and feed such as fungal strain specificity, fungal strain variation, and toxin stability [14,15,16]. However, its occurrence is closely related to environmental conditions favorable to fungal growth, in particular, a hot and humid climate, i.e., mycotoxins are most frequent in tropical areas but they can also be found in a temperate climate [17,18]. Besides that, the mycotoxin contamination may also occur at various stages of the food production chain, e.g., in the storage, in the field, during harvest, processing, and distribution [19]. Currently, the worldwide contamination of food and feedstuffs with mycotoxins is a significant problem. Based on the recent data on food grains, the prevalence of mycotoxins could be as high as 60–80%, depending on the mycotoxin of concern, the analytical method used, and the equipment detection limit [20,21].

Aflatoxins are toxic compounds produced mainly by *A. flavus* and *A. parasiticus*. Among them, aflatoxin B_1_ (AFB_1_) is the most relevant for a health concern, which has been classified as Group 1 by the International Agency for Research on Cancer (IARC) owing to its carcinogenic effect (IARC, 2012). This mycotoxin is closely associated, in long-term exposure, to the hepatocellular carcinoma in humans, modulation of the immune system and malnutrition [22].

Fumonisins are a heterogeneous group of toxins synthesized by *Fusarium* species [23]. Fumonisins of group B have been widely studied due to their prevalence and toxicological effects. In particular, fumonisin B_1_ (FB_1_) is related to chronic effects in human health, e.g., hepatocarcinoma, nephrotoxicity, suppression of the immune system and defects in the neural-tubes [24]. In addition, this mycotoxin has been classified by the IARC as a group 2B (possible carcinogen for humans) [25].

The growing consumer interest in nutritional aspects and food quality has contributed to the increased consumption of organically produced foods [26,27]. However, naturally produced and highly nutritious foods such as corn and its byproducts are also prone to fungal spoilage and mycotoxin production.

Biopreservation is a natural tool to extend shelf life and to increase the safety of foods by applying microorganisms or their antimicrobial active metabolites [28]. Recently, authors have described the use of lactic acid bacteria (LAB) to avoid the fungal growth and prolong the shelf life of food as an alternative to synthetic biocides. However, there are no reports on the use of LAB to prevent fungal growth in corn kernels and corn ears.

Previously, it was believed that the organic acids produced by the LAB were the main antifungal agents because of the lowering of the pH. However, authors have shown that the antifungal activity of these compounds alone was too low to fully explain the antifungal activity observed [29,30]. Thus, other metabolites have been studied as the source of antifungal activity such as proteinaceous compounds, fatty acids, phenolic acid, hydrogen peroxide, and bacteriocins [31,32,33,34].

It seems that the LAB possess a large potential for biopreservation due to the production of antimicrobial compounds. Moreover, it is important to emphasize that most LAB are generally recognized as safe and QPS (qualified presumption of safety), which promote them as very favorable candidates for integration as natural preservatives in food and feedstuffs [35,36]. Indeed, they are already used as starter cultures in the food industry, in dairy and dry-fermented meat products [37].

Taking into account the growing interest in the use of natural antimicrobial compounds as an alternative to synthetic chemical control and the potential of LAB, the objective of this study was to evaluate the employment of *Lactobacillus plantarum* cell-free supernatant as a biocontrol agent in order to reduce the growth of *A. flavus* ISPA 8111 (AFB_1_ producer) and *F. verticillioides* CECT 2982 (FB_1_ producer) as well as mycotoxin production on corn kernels and corn ears.

## 2. Results and Discussion

### 2.1. In Vitro Antifungal Activity of L. plantarum spp.

The qualitative antifungal effect of cell-free supernatant (CFS) obtained by *L. plantarum* spp. fermentation was determined by halo inhibition test on the solid medium of potato dextrose agar (PDA). The analyses of results demonstrated that all evaluated strains of *L. plantarum* fermented MRS, and their CFS possessed antifungal effect (Table 1). Regarding the *L. plantarum* CECT 749, its CFS showed the highest halo inhibition zone against the strain of *F. graminearum* ITEM 126, *F. cerealis* CECT 20489, *F. verticillioides* CECT 2152, *F. verticillioides* CECT 2982, and *A. flavus* ITEM 8111 in comparison to others. Moreover, the strains of *F. verticillioides* showed higher sensitivity towards *L. plantarum* CECT 749 CFS related to other fungi.

In general, the quantitative results evidenced that the *Fusarium* strains were more sensitive to CFS than *Aspergillus* strains. Moreover, agreeing to qualitative results, all CFS showed antifungal effect, presenting minimum inhibitory concentration (MIC) values ranging from 4 to 125 g/L. Specifically, the *L. plantarum* CECT 748 and *L. plantarum* CECT 749 CFS evidenced the highest antifungal activity against *Fusarium* and *Aspergillus* strains (Table 2). Furthermore, these CFS were the only ones that demonstrated fungicidal doses against *Aspergillus* strains, more precisely, the minimum fungicidal concentration (MFC) values were 125 and 62 g of CFS/L, respectively.

Analyzing the results, it is important to emphasize that the *L. plantarum* CECT 749 CFS was more efficient than others, showing the lowest concentration of MIC and MFC against all evaluated strains. Therefore, based on results, this CFS was employed in the biocontrol assays in order to avoid the growth of *A. flavus* ITEM 8111 on corn kernels and *F. verticillioides* CECT 2982 on corn ears.

### 2.2. Phenolic Acids Produced by L. plantarum spp.

**Figure 1:**
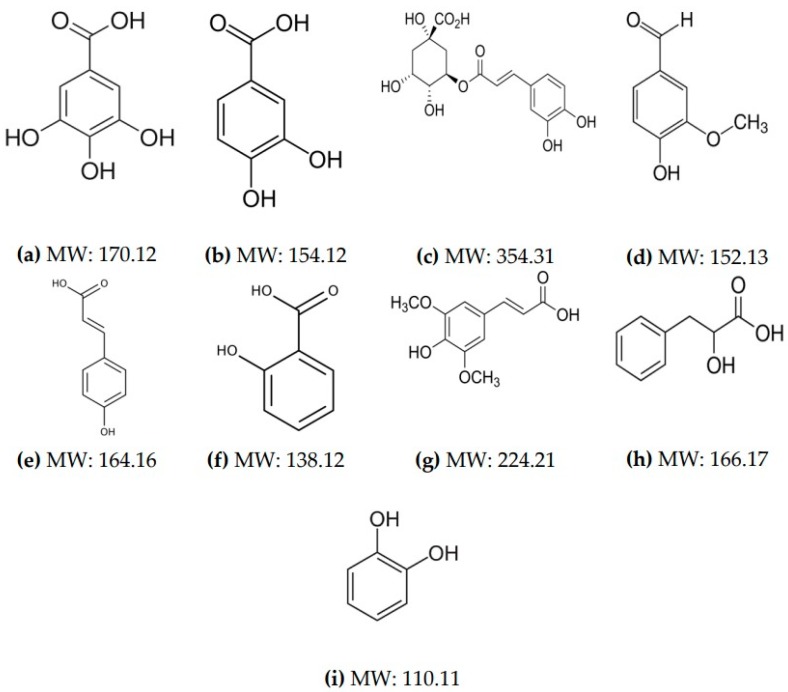
Chemical structures and molecular weight of the phenolic acids identified in the *L. plantarum* cell-free supernatant (CFS): (**a**) gallic acid; (**b**) protocatechuic; (**c**) chlorogenic acid; (**d**) vanillin; (**e**) p-coumaric acid; (**f**) salicylic acid; (**g**) sinapic acid; (**h**) phenyllactic acid; (**i**) 1,2-dihydroxybenzene; MW, molecular weight in g/mol.

In this study, nine phenolic acids were identified in MRS broth fermented by *L. plantarum* (Figure 1). These compounds were determined according to the retention time peak and molecular mass spectra obtained from the injected standard solution in LC-ESI-qTOF-MS and reported information in the literature. The phenolic acids produced by strains of *L. plantarum* are summarized in Table 3 along with their molecular formula and mass spectrum. Between the samples with the highest antifungal effect, the *L. plantarum* CECT 749 produced DL-3-phenyllactic acid, salicylic acid, and vanillin, whereas the same compounds were generated by *L. plantarum* CECT 748, in addition to chlorogenic acid, sinapic acid, and 1,2-dihydroxybenzene, and with the exception of vanillin.

Previous studies also identified phenolic acids produced by LAB in MRS broth. Rodrígues et al. noticed the presence of 1,2-dihydroxybenzene and other hydroxybenzoic acids such as vanillic acid, syringic acid, and gallic acid generated after hydrolyzation and methylation of hydroxybenzoic acid by enzymes of *L. plantarum* [38]. Brosnan et al. also identified phenolic acids for all the strains evaluated, more specifically, vanillic acid, 2-hydroxyisocapric acid, azelaic acid, decanoic acid, propanoic acid, 3-hydroxydecanoic acid, benzoic acid, hydrocinnamic acid, DL-p-hydroxyphenyllactic acid, phenyllactic acid, DL-β-hydroxylauric acid, 4-hydroxybenzoic acid, 3-4-hydroxy-3-methoxyphenyl, and 1,2-dihydroxybenzene [39]. Indeed, it is known that *L. plantarum* synthesizes phenolic acids by decarboxylation and/or reduction [40]. These compounds can act as chaotropic stressors, causing a disorder and cellular stress with the consequent lysis of the plasma membrane [41].

Some phenolic acids—above described—were earlier studied as antifungal compounds. Among them, the 1,2-dihydroxybenzene—often known as pyrocatechol or catechol—is commonly found in its bound form in foods as part of complex structures such as lignins and hydrolyzable tannins. In addition, this phenolic acid was previously described as an antifungal compound [38,42].

Silva et al. assessed the correlations between phenolic compounds and their ability to avoiding aflatoxin production on soybeans. They demonstrated that phenolic compounds such as vanillin were able to contribute to a defense mechanism against fungal spoilage and AFB_1_ production depending on the level of contamination [43]. In addition, Romero-Cortes et al. evaluated the antifungal activity of vanillin against *Alternaria alternata* isolated from sorghum and barley. The vanillin showed a fungicidal effect in a dose-dependent manner; moreover, doses lower than 750 mg/L of vanillin reduced the fungal growth, increasing the lag phase from 50 to 112 h [44].

Saladino et al. evaluated the antifungal activity of LAB strains against *A. parasiticus*. The authors described that the fermentation of bread dough with LAB reduced the fungal growth as well as the content of aflatoxins. They also attributed the effectiveness of LAB by phenolic compounds and bioactive peptides presented in the media and the lowering of pH [28]. Our results pointed out that the *L. plantarum* CECT 220, CECT 221 and CECT 748 showed the highest capacity to produce phenolic compounds. And, the other strains of *L. plantarum* produced at least two phenolic compounds. In addition, it is important to highlight that the phenolic acids identified in this study were in agreement with those that have been detected by other authors.

Despite the fact that the phenolic acids found in the CFS have previously been reported as antifungal compounds, the antifungal and antimycotoxigenic effects evidenced in our study could be attributed to the bio-complex obtained by *L. plantarum* MRS fermentation. This bio-complex could contain other metabolites such as proteins, peptides, and organic acids that are widely known as antifungal compounds but have not been identified in our study [28,33,45]. In other words, it seems that the presence of phenolic acids in the CFS only contributed to enhancing the antifungal activity of the bio-complex.

### 2.3. L. plantarum CECT 749 CFS Characteristics and Composition

*L. plantarum* CECT 749 was incubated on MRS Broth for 72 h at 37 °C. After that, the bacteria cells were separated from the liquid fraction by centrifugation and, the CFS was recovered as described in Section 4.3. The methodology and composition of *L. plantarum* CECT 749 CFS are plotted in Table 4. The organic acids and the final pH of the solution play an important role in mycelial growth [46]. Gerez et al. evaluated the antifungal activity of lactic acid, acetic acid, phenyllactic acid, and propionic acid against *Aspergillus niger*, *Fusarium graminearum*, and *Penicillium* spp. under pH 3.5 and 6.0. These authors reported that the lowest concentration of organic acid -necessary to produce a 50% inhibition of conidia germination after 48 h of incubation was significantly reduced in pH 3.5. For instance, the MIC of lactic acid at pH 3.5 ranged from 2.5 to 180 mM whereas in pH 6.0 the MIC ranged from 50 to 300 mM [46]. Our results corroborate with those since the pH of 3.3 ± 0.3, as well as the high content of lactic acid (15 g/L) could also contribute to the antifungal activity of the CFS.

### 2.4. Biopreservation and Shelf-Life Improvement by L. plantarum CECT 749 CFS

In addition to evaluating the qualitative and quantitative antifungal activity of CFS, the ability to enhance shelf life was investigated on corn kernels and corn ears. The shelf life was determined by the first sign of visual fungal growth in the matrices.

The fungal growth of the control group under similar storage conditions was noticed on corn kernels after seven days, whereas on corn ears the shelf life was determined on the fifth day onwards. Compared to control, the spray of *L. plantarum* CECT 749 CFS delayed the growth of both *A. flavus* and *F. verticillioides* CECT 2982 in eight and two days, respectively (Table 5). In addition, it is noteworthy that the samples of corn kernels and corn ears were treated, dried, and contaminated with final moisture content (MC) of 18 and 20%, respectively. Thus, the lower reduction in shelf life noticed on corn ears could be explained by the higher MC.

These results suggested that the CFS may be used as a biocontrol to reduce *F. verticillioides* growth on corn ears and *A. flavus* growth on corn kernels during storage, even under inappropriate MC condition.

**Figure 2:**
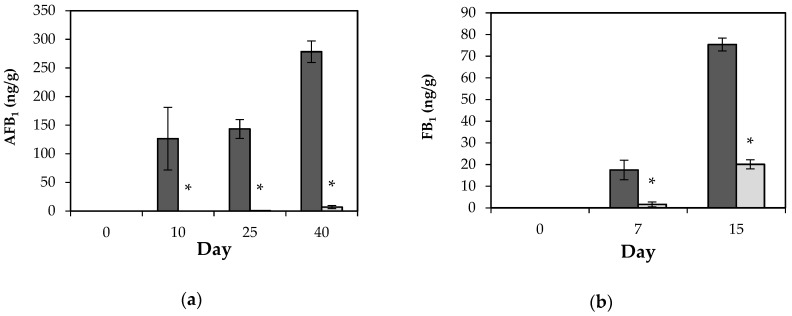
Effect of *L. plantarum* CECT 749 CFS on mycotoxin production: (**a**) AFB_1_ content (ng/g) in corn kernels contaminated with *A. flavus* ITEM 8111 at different storage times (0, 10, 25 and 40 days). Control samples (dark grey) and treated samples (clear grey); (**b**) FB_1_ content (ng/g) in corn ears contaminated with *Fusarium verticillioides* CECT 2982 at different storage times (0, 7 and 15 days). Control samples (dark grey) and treated samples (clear grey). (*) Represents a significant difference among the treatments (*p* ≤ 0.05). The experiment was carried out twice with analyses in triplicate (n = 6).

Corn is a rich substrate for aflatoxin production and earlier reports demonstrated the effective use of antifungal properties of bioactive compounds produced by LAB for inhibiting AFB_1_ in various crops [51]. In our study, the *L. plantarum* CECT 749 CFS reduced the fungal growth, hence, the AFB_1_ production by 100% at day 5. In addition, applying the CFS spray on the surface of corn before contamination reduced the AFB_1_ production by 99.7 and 97.5% at days 25 and 40, respectively (Figure 2). According to visual FG analyses, the *L. plantarum* CECT 749 CFS did not avoid the AFB_1_ production, but this preventive treatment inhibited its production by 10 days.

The data related to FB_1_ production are plotted in Figure 2. The results showed that *L. plantarum* CECT 749 CFS possess antimycotoxigenic activity against *F. verticillioides* CECT 2982, reducing the FB_1_ production by 90.6% at day 7. However, it was realized that the antimycotoxigenic effect provoked by treatment decreased over time, reaching to 73.7% of reduction after 15 days of storage. The mycotoxin reduction, evidenced in this study, could be explained perhaps because the treatment with CFS delayed the fungal growth, hence, the fungi need more time to achieve the secondary metabolism.

Authors have noticed that LAB have the potential to either bind and mitigate mycotoxin or, to some extent, inhibit the fungal growth [52,53,54,55]. Although the LAB antimycotoxigenic mechanisms of action have not yet been explained, analyzing our data, it seems that they may be closely linked to the antifungal effects evidenced in this work. Damayanti et al. isolated twenty-eight LAB from cassava and bean fermentation. The LAB with the highest activities was *L. plantarum* G7 [56]. Its CFS extract showed inhibition of mycelial growth between 39–63.1%. Consequently, the aflatoxin content was reduced. In addition, Russo et al. investigated the antifungal capacity of eighty-eight *L. plantarum* strains [57]. They concluded that the employment of LAB increased shelf life. Although they had investigated the antifungal activity of CFS against toxigenic fungi, the mycotoxin content was not evaluated in the food samples.

Overall, the treatment did not completely avoid AFB_1_ and FB_1_ production, however, in both corn kernels and corn ears, the final concentration of mycotoxin was significantly reduced after fumigation of *L. plantarum* CECT 749 CFS compared to the control group. For instance, after incubation time, the final AFB_1_ and FB_1_ concentration in the treatment group was 6.9 and 20.1 ng/g, whereas the concentration of AFB_1_ and FB_1_ in the control group reached values of 278.4 and 75.4 ng/g, respectively.

On the one hand, despite the large reduction of AFB_1_ provoked by CFS application, the levels of AFB_1_ were higher than those set by the European Union (5 ng/g). On the other hand, the levels of AFB_1_ and FB_1_ were lower than those established by the FDA (20 ng/g of AFB_1_ and 2000 ng/g of FB_1_) to non-processed corn for human consumption [58,59,60].

The present results suggested that metabolism products of LAB, due to their potential to reduce the growth of the mycotoxigenic fungi and the biosynthesis of the mycotoxins, could be promising for the biocontrol of grains such as corn.

Corn grains are constantly stored for a long time before commercialization, further studies could be performed, increasing samples analysis time and setting the MC at a percentage slightly over 14% since this percentage is adequate for grain storage [61]. In addition, we expect to isolate the antifungal compounds produced by the *L. plantarum* CECT 749 and evaluate the purified fraction activity as well as to compare them to the whole CFS extract. In addition, it might be worthwhile to increase the experiment from a laboratory scale to an industrial scale in order to produce more compounds and to evaluate their constancy of production.

## 3. Conclusions

In the past, LAB have shown potential as antifungal agents to avoid fungal spoilage and the related mycotoxin production in foods such as dairy and fermented products. In this study, in vitro analyses have shown that *Fusarium* strains were more susceptibility to CFS treatment than *Aspergillus* spp. In terms of fungal growth and mycotoxin production in both corn kernel and corn ears, the CFS treatment did not completely inhibit either the fungal growth or mycotoxin production. Probably, high levels of antifungal compounds associated with lower MC might be required to completely avoid the growth of *A. flavus* and *F. verticillioides* on corn kernels and corn ears. In contrast, even when the mycotoxin production occurred, the content of FB_1_ and AFB_1_ was significantly reduced. Therefore, these results provide new insights for the biotechnological employment of *L. plantarum* CECT 749 CFS for biopreservation of corn kernels and corn ears.

## 4. Materials and Methods

### 4.1. Chemicals

AFB_1_ and FB_1_ standard solutions (purity > 99%) were purchased from Sigma-Aldrich (St. Louis, MO, USA). The phenolic compounds gallic acid, sinapic acid, chlorogenic acid, caffeic acid, syringic acid, vanillin, vanillic acid, hydroxybenzoic acid, hydroxylcinnamic acid, p-coumaric acid, benzoic acid, DL-3-phenyllactic acid, 1,2-dihydroxybenzene, 3,4-dihydroxyhydrocinnamic, and DL-p-hydroxyphenyllatic acid were provided from Sigma-Aldrich (St. Louis, MO, USA). Phenyllactic acid was obtained from BaChem (Weil am Rhein, Germany). Ferulic acid was purchased from MP Biomedicals (Santa Ana, CA, USA) and protocatechuic acid from HWI pharma services (Ruelzheim, Germany). Acetonitrile (ACN) (99%), methanol (99%), ethyl acetate (EA) (99%) and formic acid (FA) (99%) used for liquid chromatography were of HPLC-grade and obtained from VWR Chemicals (Radnor, PA, USA). Magnesium sulfate (MgSO_4_), ammonium formate and sodium chloride (NaCl) were obtained from Sigma-Aldrich (St. Louis, MO, USA). Sepra^TM^ C18-E (50 μm, 65 Å) was purchased from Phenomenex (Madrid, Spain). Microbiological media such as potato dextrose broth (PDB), potato dextrose agar (PDA), Man Rogosa Sharpe (MRS) broth and MRS Agar were obtained from Liofilchem Bacteriology Products (Roseto degli Abruzzi, Italy). Deionized water (<18 MΩ cm resistivity) was obtained from a Milli-Q purification system (Millipore, Bedford, MA, USA).

### 4.2. Microorganisms and Culture Conditions

Fungal strains *A. parasiticus* CECT 2681, *A. niger* CECT 2088, *F. verticillioides* CECT 20926, *F. verticillioides* CECT 2152, *F. verticillioides* CECT 2982, *F. cerealis* CECT 20488, *F. cerealis* CECT 20489, *F. mesoamericanum* CECT 20490, and *F. poae* CECT 20,165 were obtained from the Spanish Type Culture Collection CECT (Valencia, Spain). *F. graminearum* ITEM 126, *F. graminearum* ITEM 6415 and *A. flavus* ITEM 8111 were obtained from the microbial culture collection of the Institute of Sciences of Food Production, ISPA (Bari, Italy). These microorganisms were maintained in sterile glycerol (30%) at −80 °C prior to analysis. The fungal strains were cultivated on PDB, transferring 1 mL of the glycerinated solution to 9 mL of PDB medium. The contaminated PDB was incubated for 72 h at 25 °C, and then, aliquots were plated on PDA Petri dishes in order to obtain spores. These spores were used to contaminate the corn samples.

LAB strains *Lactobacillus plantarum* CECT 220, *L. plantarum* CECT 221, *L. plantarum* CECT 223, *L. plantarum* CECT 224, *L. plantarum* CECT 748, *L. plantarum* CECT 749 and *L. plantarum* CECT 750 were obtained from the Spanish Type Culture Collection CECT (Valencia, Spain). The LAB were kept in frozen glycerol (3%) stocks at −80 °C until analysis. Prior to fermentation assays, 1 mL of LAB glycerinated was homogenized with 9 mL of MRS Broth culture medium and incubated under anaerobic conditions for 48 h at 37 °C.

### 4.3. LAB Growth and Preparation of Cell-Free Cupernatant

LAB were incubated on MRS Broth for 12 h at 37 °C in order to reach the exponential growth phase. Then, 10 mL of incubated MRS Broth at a concentration of 10^7^ CFU/mL were placed in other Falcon tubes containing 40 mL of sterile MRS Broth. Subsequently, the tubes were incubated under anaerobic conditions for 72 h at 37 °C to allow for MRS fermentation. After that, LAB cells were separated from the fermented solution by centrifugation at 4000× *g* for 10 min. Lastly, cell-free supernatants (CFS) were recovered and freeze at −80 °C for 24 h before drying with a lyophilizer FreeZone 2.5 L Labconco (Kansas, MO, USA). The lyophilized CFS was stored at −18 °C prior to antifungal analyses.

### 4.4. Qualitative Antifungal Activity in Solid Medium

The antifungal effect of lyophilized CFS was evaluated towards strains of *Aspergillus* and *Fusarium* genera.

First, Petri dishes containing PDA were contaminated with harvested fungal conidia previously cultivated on PDA plates, as described by Section 4.2. The conidia were scraped from agar and dispersed on another PDA plate, using sterile cotton swabs. Briefly, 10 mm diameter wells were made using sterile pipette tips, whereas each well was filled with 50 µL of lyophilized CFS which was previously resuspended in sterile water at the concentration of 250 g/L. A sterile water solution containing lyophilized MRS Broth was used as negative control. After that, the PDA plates were incubated for 72 h at 25 °C to allow the fungal growth and diffusion of the CFS through agar. Finally, the antifungal effect was determined by measuring the inhibition zone around the well. The treatments which showed an inhibition zone larger than 7 mm between the well and fungal growth were considered positive for antifungal activity [62].

### 4.5. Quantitative Antifungal Activity in 96-Well Microplates

The quantitative assay was performed as described by the guidelines in CLSI document M38-A2 with slight modifications [63]. First, 100 μL of PDB containing lyophilized CFS at concentrations from 0.48 to 500 g/L was added to 96-well sterile microplates. Then, 100 μL of PDB containing 5 × 10^3^ conidia of toxigenic fungi were added to each well. The positive control consisted of contaminated PDB medium with lyophilized non-fermented CFS (250 g/L) and the negative control was prepared with a non-contaminated PDB medium without any treatment. Posteriorly, the microplates were incubated at 25 °C for 3 days.

The MIC was defined as the lowest concentration of the fermented CFS that did not show any visible growth. Four replicates of each treatment were carried out. After that, 10 μL of the concentration corresponding to the MIC and higher concentrations evaluated were recultivated on PDA plates for the determination of the MFC. Plates were incubated at 25 °C for 72 h and the MFC was determined as the concentration that avoided any visible fungal growth.

### 4.6. Extraction and Identification of Phenolic Acids by LC-ESI-qTOF-MS

The CFS extracts were cleared by QuEChERS methodology prior to chromatographic analysis as described by Brosnan et al. [64]. Thus, ten milliliters of fermented CFS were extracted with 10 mL ethyl acetate 1% formic acid, 4 g of MgSO_4_, 1 g of NaCl and then vortexed for 1 min. The extract was centrifuged at 4000× *g* for 10 min, and the supernatant was recovered in a Falcon tube containing 150 mg of C18 and 900 mg MgSO_4_. The extraction was realized twice and the supernatant was evaporated under nitrogen flow. Afterward, the dried supernatant was resuspended in 1 mL of H_2_O: ACN (90:10) prior to chromatographic analysis.

The LC system used for the chromatographic determination was an Agilent 1200 (California, USA) equipped with a vacuum degasser, autosampler, and binary pump. The stationary phase was a Phenomenex column (Gemini C18 50 × 2 mm, 100 Å and particle size of 3 μm). Mobile phases consisted of water as solvent A, ACN as solvent B both acidified (0.1% formic acid) and the gradient elution was established as follows: 0 min, 5% B; 30 min 95% B; 35 min, 5% B. The column was equilibrated for 3 min prior to analyses. The flow rate used was 0.3 mL/min and the sample volume injected was 20 μL.

Mass spectrometry analysis was carried out using a Q-TOF-MS (6540 Agilent Ultra High Definition Accurate Mass) equipped with an Agilent Dual Jet Stream electrospray ionization (Dual AJS ESI) with interface in negative ionization mode at the following conditions: drying gas flow (N_2_), 8.0 L/min; nebulizer pressure at 30 psig; gas drying temperature at 350 °C; capillary voltage at 3.5 kV; fragmentor voltage and scan range were 175 V and m/z 20–380, respectively. Targeted MS/MS experiments were carried out using collision energy of 10, 20 and 40 eV. Integration and data elaboration were realized using Masshunter Qualitative Analysis Software B.08.00 [65].

### 4.7. Biopreservation of Corn Kernels and Corn Ears

To evaluate the antifungal and antimycotoxigenic activity of the CFS by *L. plantarum* CECT 749, the study was carried out using corn kernels contaminated with *A. flavus* ISPA 8111 (AFB_1_ producer) and corn ears contaminated with *F. verticillioides* CECT 2982 (FB_1_ producer). These strains were selected according to the fungal sensitivity to fermented CFS—demonstrated by qualitative and quantitative assays (Section 4.4 and Section 4.5); the main steps of food production chain in which the fungal contamination occurs; and the mycotoxin production—previously determined in corn and corn ears.

The biopreservation test was performed in laboratory scale silos as described by Nazareth et al. with modifications [60].

**Figure 3:**
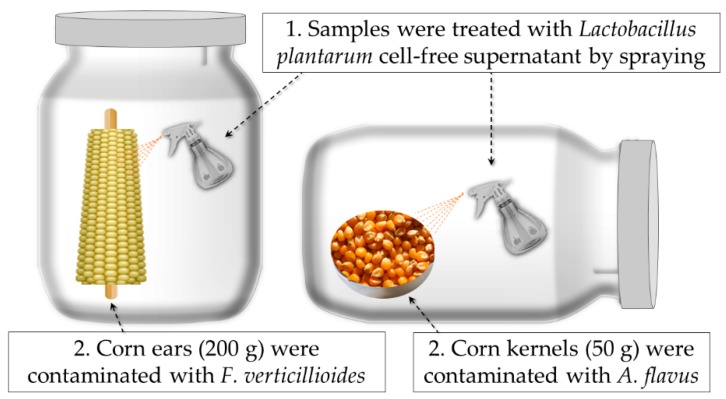
Biopreservation test of corn ears and corn kernels in a lab system. The samples were treated with *Lactobacillus plantarum* CECT 749 cell-free supernatant. After drying, corn kernels were contaminated with *Aspergillus flavus* ISPA 8111 and corn ears were contaminated with *Fusarium verticillioides* CECT 2982.

Samples of corn kernels (50 g) were divided into two groups: a group was sprayed with 10 mL of lyophilized CFS in order to obtain a final concentration of 12.5 mg of CFS/g of corn, and a control group was sprayed with 10 mL of sterile water (Figure 3). Next, the corn kernels were dried in an incubator (Memmert D06061 Modell 500; Memmert GmbH + Co. KG, Schwabach, Germany) at 50 °C for 30 min. Then, both groups were inoculated with suspensions containing 1 mL of *A. flavus* ISPA 8111 at 10^3^ conidia/g. Lastly, the sampling was performed on days 0, 7, 15, 40 to determine the AFB_1_ production.

Similarly, 200 g of corn ears were treated with 6 mL of CFS equivalent to a concentration of 1.6 mg of lyophilized CFS/g of corn ears. In this case, corn ears were contaminated with 1 mL of *F. verticillioides* CECT 2982 suspension at a concentration of 10^3^ conidia/g. Further, the FB_1_ production was determined on days 0, 7 and 15. Moreover, visual fungal growth was monitored daily in both studies. In addition, samples were placed in the incubator at 105 °C for 24 h and the initial MC was measured by gravimetric methodology [66].

### 4.8. Determination of Mycotoxins by LC-MS/MS Spectrometry

AFB_1_ and FB_1_ mycotoxins were extracted using the method described and validated by Quiles et al. [67]. Corn kernels were ground with an Oester Classic grinder (Madrid, Spain) in order to reduce particle size. Corn ears were frozen at −80 °C and lyophilized before grinding. The samples were weighed in 5 g, placed in a Falcon tube with 25 mL of MeOH, and homogenized for 3 min using an Ultra Ika T18 basic ultraturrax (Staufen, Germany). Following this, the extract was centrifuged at 4000× *g* for 15 min at 4 °C, and the supernatant was evaporated to dryness with Büchi Rotavapor R-200 (Postfach, Switzerland). Finally, the dried extracts were resuspended in 2 mL of methanol and filtered in 0.22 µm before LC-MS/MS analysis.

The liquid-chromatography system consisted of an LC-20AD pump coupled to a 3200QTRAP mass spectrometer (Applied Biosystems, Foster City, CA, USA) using an ESI interface in positive ion mode. The mycotoxins were separated on a Gemini NX C18 column 150 × 2 mm × 3 µm Phenomenex (Palo Alto, CA, USA). The mobile phases were the solvent A (5 mM ammonium formate and 0.1% formic acid in water) and solvent B (5 mM ammonium formate and 0.1% formic acid in methanol) at a flow rate of 0.25 mL/min. The elution was carried out using a linear gradient from 0 to 14 min. The injection volume set was 20 µL, the nebulizer, the auxiliary and the auxiliary gas were set at 55, 50, and 15 psi respectively. The capillary temperature and the ion spray voltage were 550 °C and 5500 V, respectively. The ions transitions used for the mycotoxins identification and quantification were: m/z 313.1, 241.3, and 284.9 for AFB_1_, m/z 352.4 and 334.4 for FB_1_.

### 4.9. Statistical Analysis

The software Prism version 3.0 (GraphPad, La Jolla, CA, USA) for Windows was used for the statistical analysis of data. The experiments were realized in triplicates and the difference between control and treated group was analyzed by Student’s t-test. The level of significance was considered as *p* ≤ 0.05.

## Figures and Tables

**Table 1:** Antifungal activity evaluated in potato dextrose agar (PDA) solid medium and treated with *Lactobacillus plantarum* cell-free supernatant (CFS) at 250 g/L against toxigenic fungi of *Aspergillus* and *Fusarium* genera. The antifungal activity was expressed as follows: (+) means 8 mm of inhibition zone between the well and fungal growth, (+ +) means 8–10 mm of inhibition zone between the well and fungal growth, (+ + +) means > 10 mm of inhibition zone between the well and fungal growth.

Fungal Strain	*L. plantarum*						
	CECT 220	CECT 221	CECT 223	CECT 224	CECT 748	CECT 749	CECT 750
*F. graminearum* ITEM 126	+	+	+	+	+	+ + +	+
*F. graminearum* ITEM 6415	+	+	+	+	+	+	+
*F. cerealis* CECT 20488	+	+	+	+	+	+	+
*F. cerealis* CECT 20489	+	+	+ +	+	+	+	+
*F. verticillioides* CECT 20926	+	+	+	+	+ +	+ + +	+
*F. verticillioides* CECT 2152	+ +	+	+ +	+	+ +	+ + +	+ +
*F. verticillioides* CECT 2982	+ +	+ +	+ +	+	+ +	+ + +	+
*F. mesoamericanum* CECT 20490	+	+	+	+	+	+	+
*F. poae* CECT 20165	+	+	+	+	+	+	+
*A. flavus* ITEM 8111	+	+	+	+	+	+ +	+
*A. parasiticus* CECT 2681	−	−	−	−	+	+	−
*A. niger* CECT 2088	−	+	−	−	+	+	+

**Table 2:** Minimum inhibitory concentration (MIC) and minimum fungicidal concentration (MFC) expressed in g/L of *Lactobacillus plantarum* cell-free supernatants (CFS) against *Aspergillus* and *Fusarium* strains.

Fungal Strain	*L. plantarum*													
	CECT 220		CECT 221		CECT 223		CECT 224		CECT 748		CECT 749		CECT 750	
	MIC	MFC	MIC	MFC	MIC	MFC	MIC	MFC	MIC	MFC	MIC	MFC	MIC	MFC
*F. graminearum* ITEM 126	8	8	8	16	8	16	8	16	8	8	8	16	8	16
*F. graminearum* ITEM 6415	8	16	8	13	8	13	8	13	4	16	4	16	4	16
*F. cerealis* CECT 20488	8	16	8	31	8	16	8	16	8	8	8	16	8	16
*F. cerealis* CECT 20489	8	16	16	16	16	31	4	16	4	16	4	16	4	31
*F. verticillioides* CECT 20926	16	31	16	31	16	31	16	31	8	16	8	31	16	31
*F. verticillioides* CECT 2152	4	31	4	31	4	31	4	31	4	31	4	31	4	31
*F. verticillioides* CECT 2982	16	31	16	31	16	31	16	31	16	31	16	31	16	31
*F. mesoamericanum* CECT 20490	8	16	8	31	8	16	8	16	8	16	8	16	8	16
*F. poae* CECT 20165	16	31	8	16	8	16	8	16	8	16	8	16	8	16
*A. flavus* ITEM 8111	250	nd ^1^	250	nd ^1^	250	nd ^1^	250	nd ^1^	125	250	62	125	250	nd ^1^
*A. Parasiticus* CECT 2681	nd ^1^	nd ^1^	nd ^1^	nd ^1^	nd ^1^	nd ^1^	nd ^1^	nd ^1^	250	nd ^1^	125	250	250	nd ^1^
*A. niger* CECT 2088	nd ^1^	nd ^1^	250	nd ^1^	nd ^1^	nd ^1^	nd ^1^	nd ^1^	250	nd ^1^	250	nd ^1^	250	nd ^1^

^1^ nd = non-detected.

**Table 3:** Phenolic compounds quantified in the cell-free supernatant (CFS) of *Lactobacillus plantarum* strains by LC-ESI-qTOF-MS.

Compound	Molecular Formula	*L. plantarum*						
		CECT 220	CECT 221	CECT 223	CECT 224	CECT 748	CECT 749	CECT 750
Gallic acid	C_7_H_6_O_5_	0.7 ± 0.2	nd ^1^	nd ^1^	nd ^1^	nd ^1^	nd ^1^	nd ^1^
Protocatechuic	C_7_H_6_O_4_	nd ^1^	nd ^1^	0.4 ± 0.1	nd ^1^	nd ^1^	nd ^1^	nd ^1^
Chlorogenic acid	C_16_H_18_O_9_	0.6 ± 0.1	nd ^1^	nd ^1^	nd ^1^	0.5 ± 0.1	nd ^1^	nd ^1^
Vanillin	C_8_H_8_O_3_	nd ^1^	0.3 ± 0.1	nd ^1^	nd ^1^	nd ^1^	0.5 ± 0.2	nd ^1^
p-coumaric acid	C_9_H_8_O_3_	nd ^1^	0.3 ± 0.1	nd ^1^	0.6 ± 0.1	nd ^1^	nd ^1^	nd ^1^
Salicylic acid	C_7_H_6_O_3_	0.4 ± 0.1	0.3 ± 0.1	0.5 ± 0.2	0.3 ± 0.1	0.6 ± 0.2	0.5 ± 0.1	0.6 ± 0.1
Sinapic acid	C_11_H_12_O_5_	0.7 ± 0.1	nd ^1^	nd ^1^	nd ^1^	1.0 ± 0.2	nd ^1^	0.4 ± 0.1
Phenyllactic acid	C_9_H_10_O_3_	1.0 ± 0.2	1.1 ± 0.1	0.9 ± 0.1	1.4 ± 0.2	3.9 ± 0.1	5.3 ± 0.3	2.8 ± 0.2
1,2-Dihydroxybenzene	C_6_H_6_O_2_	nd ^1^	0.5 ± 0.1	nd ^1^	0.6 ± 0.2	1.1 ± 0.2	nd ^1^	0.9 ± 0.2

^1^ nd = non-detected. Mean ± standard deviation (n = 6).

**Table 4:** Characteristics and composition of cell-free supernatant (CFS) produced by *L. plantarum* CECT 749.

Parameters	*L. plantarum* CECT 749 CFS (%)	MRS (%)
Moisture [47]	94.5 ± 2.10	94.3 ± 2.40
Proteins [46]	0.3 ± 0.07	2.5 ± 0.30
Ash [48]	0.2 ± 0.06	0.2 ± 0.01
Carbohydrates [49]	3.2 ± 0.31	2.7 ± 0.5
pH	3.3 ± 0.21	6.2 ± 0.22
Lactic acid [50]	1.5 ± 0.50	nd ^1^

^1^ nd = non-detected. Mean ± standard deviation (n = 6).

**Table 5:** Fungal growth monitored in corn kernels contaminated with *Aspergillus flavus* ITEM 8111 and corn ears contaminated with *Fusarium verticillioides* CECT 2982 during the storage period. Fungal growth is expressed as (+) and absence of fungal growth as (−).

Samples	*A. flavus* ITEM 8111				*F. verticillioides* CECT 2982			
	Days							
	0	7	15	40	0	5	7	15
Control	−	+	+	+	−	+	+	+
CFS	−	−	+	+	−	−	+	+
